# Bridge of Tunneled Cuffed Catheter as a Risk for Future Arteriovenous Fistulae Failure

**DOI:** 10.3390/jcm11051289

**Published:** 2022-02-26

**Authors:** Chung-Kuan Wu, Yen-Chun Huang, Chia-Hsun Lin, Mingchih Chen

**Affiliations:** 1 Division of Nephrology, Department of Internal Medicine, Shin Kong Wu Ho-Su Memorial Hospital, Taipei 11101, Taiwan; m008533@ms.skh.org.tw; 2 School of Medicine, Fu Jen Catholic University, New Taipei City 24205, Taiwan; chlin7667@yahoo.com.tw; 3 Dialysis Vascular Access Management Center, Shin Kong Wu Ho-Su Memorial Hospital, Taipei 11101, Taiwan; 4 AI Development Center, Fu Jen Catholic University, New Taipei City 24205, Taiwan; hivicky92@gmail.com; 5 Graduate Institute of Business Administration, College of Management, Fu Jen Catholic University, New Taipei City 24243, Taiwan; 6 Division of Cardiovascular Surgery, Department of Surgery, Shin Kong Wu Ho-Su Memorial Hospital, Taipei 11101, Taiwan

**Keywords:** NHIRD, tunneled cuffed catheter, bridge, arteriovenous fistula failure

## Abstract

Background: A clinically tunneled cuffed catheter (TCC) for hemodialysis (HD) is often inserted into end-stage renal disease patients, who have an immature or no arteriovenous fistula (AVF), for the performance of HD to relieve uremic syndrome or to solve uncontrolled fluid overload, hyperkalemia, or metabolic acidosis. The catheter is primarily regarded as a bridge until the AVF matures and can be cannulated for HD. However, the effect of the bridge of the TCC on the future patency of AVFs remains elusive. Methods: This nationwide population-based observational study compared the hazards of AVF failure and the time to AVF failure. We enrolled 24,142 adult incident patients on HD, who received HD via AVFs for at least 90 days between 1 January 2010 and 31 December 2015. The subjects were divided into two groups, according to the history of TCC, and were followed-up until the failure of the AVF, mortality, or the end of the study. A propensity score-matched analysis based on 1:1 matching of age, sex, and baseline comorbidities was utilized to reduce bias and confounding variables. Results: A Kaplan–Meier survival curve revealed that patients with and without a history of TCC had significantly better AVF survival rates (log-rank test; *p* < 0.001). A history of TCC was independently associated with a higher risk of new AVF or AVG creation due to AVF failure, after the adjustment of the Charlson comorbidity index score (corresponding adjusted hazard ratios of 2.17 and 1.52; 95% confidence intervals of 1.77–2.67 and 1.15–1.99). For the impact of time on AVF failure, patients with a TCC bridge had a significantly higher incidence of new AVF creation during the first year after the AVF cannulation. Conclusion: A history of a TCC bridge was an independent risk factor for AVF failure and the time of AVF failure was significantly higher during the first year after the fistula cannulation in the TCC bridge group.

## 1. Introduction

Vascular access (VA) is the lifeline for hemodialysis (HD) patients, and efficient HD is dependent on well-functioning VA. Three main types of VA, namely, native arteriovenous fistulae (AVF), arteriovenous grafts (AVG), and tunnel-cuffed catheters (TCC), can support patients for long-term HD. An AVF is considered the best VA option when feasible because of its longevity, lower infection risks, and fewer long-term VA events, such as thrombosis, and interventions [1,2]. A TCC is considered as the last choice and should be avoided because of its high morbidity and mortality [3].

KDOQI guidelines recommended that referral for dialysis access creation should be made when CKD patients had an eGFR of 15–20 mL/min/1.73 m^2^, and ESVS recommended that permanent VA should be created 3–6 months before the expected start of HD treatment [4,5]. However, in most clinical scenarios, ESRD patients with uremic syndrome or uncontrolled renal complications need dialysis, but do not have any VA for HD or their AVF is immature for use. Therefore, using a TCC for HD in these clinical circumstances is reasonable. For these patients, who start with a TCC for HD without any VA, a dialysis access plan should be created within 30 days from the start of the dialysis [1]. For patients with immature AVF, a TCC should be used in the short-term as a bridge for HD until the maturation of the AVF. 

In the United States, the Fistula First Breakthrough Initiative (FFBI) was conceived in 2003 to help the Institute for Healthcare Improvement. The prevalence of AVF significantly increased, but prevalent TCC use remained unchanged in the mid–20% range since the FFBI [6]. This phenomenon may be explained by several factors. First, more TCC served as a bridge access after the FFBI. Second, the failure of AVF remained high, which delayed the conversion of TCC to AVF [7]. In Taiwan, the ratio of adult incident dialysis with created VA to initiate the first HD is approximately 40%. Among these patients, the ratio can reach 58% for patients attending the preESRD program but only 23% for those who do not attend such program [8]. Accordingly, the use of the TCC as a bridge for HD in Taiwan remains high. Therefore, this study aimed to investigate the effect of the bridge of the TCC on future AVF outcome. This work was also designed to compare the AVF abandonment rate and the time of the AVF abandonment for adult patients via functional AVF for HD, with or without a history of a TCC used as a bridge.

## 2. Materials and Methods

### 2.1. Data Sources and Research Samples

The National Health Insurance (NHI) system in Taiwan was built in 1995, and approximately 23 million individuals joined this program. The NHI Research Database (NHIRD) provides information on healthcare utilization and gradually collects each patient’s visit records. The diagnosis code is identified using the International Classification of Diseases, Ninth and Tenth Revision, Clinical Modification (ICD-9-CM, ICD-10-CM). To protect personal information and follow the secrecy guidelines, the patient’s personal information and ID are all anonymous in the NHIRD. This study was approved, and informed consent was waived by the Institutional Review Board of Fu Jen Catholic University in Taiwan (IRB Approval No. C104016).

### 2.2. Study Population and Exclusion Criteria

Patients were selected from the NHIRD, which included patients with new regular HD, and patients who had AVG or AVF (*n* = 33,300) between 1 January 2010 and 31 December 2015. The index date was defined as the first HD date. This research excluded the following criteria: age ≤ 18 years (*n* = 25), missing information (*n* = 16), kidney transplant (*n* = 38), conversion from HD to PD (*n* = 152), death within 1 year (*n* = 1613), and AVG patients (*n* = 7314).

**Figure 1:**
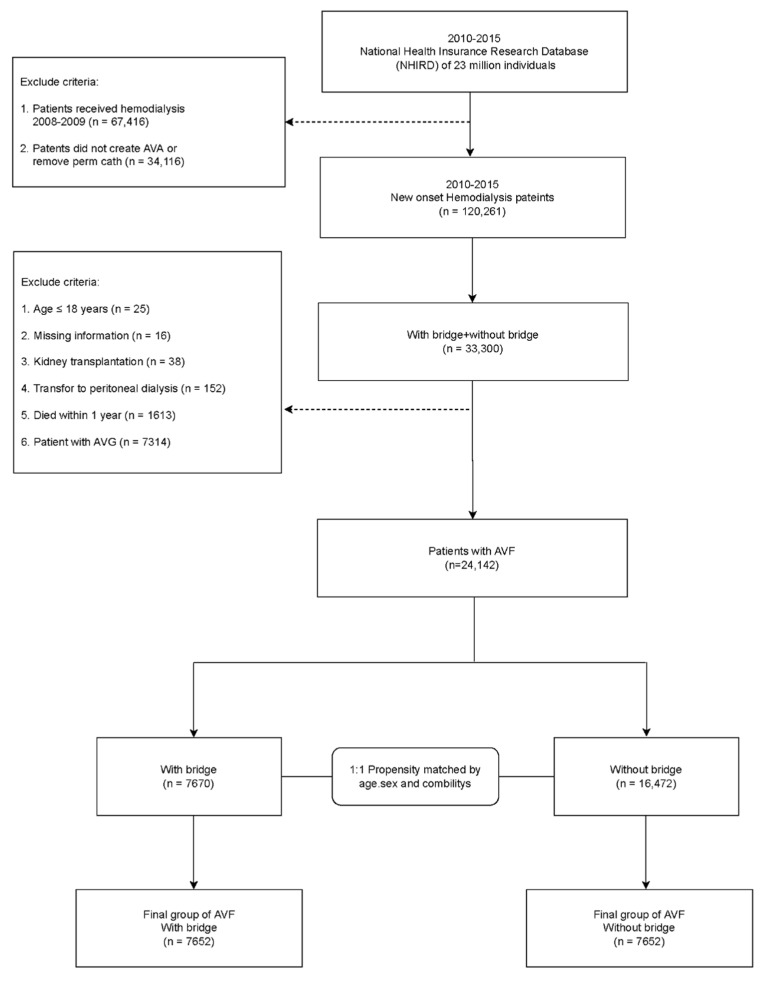
Flow chart of patient selection for the study cohort.

Finally, we divided the AVF patients into two groups, namely, with a bridge (*n* = 7670) and without a bridge (*n* = 16,472) as shown in Figure 1. To reduce the difference between the groups, we used propensity score-matched analysis based on 1:1 matching of age, sex, and baseline disease, which included peripheral artery disease (PVD), chronic obstructive pulmonary disease (COPD), coronary heart disease (CAD), hyperlipidemia, hypertension, diabetes, and stroke. After matching, for the final group of AVF patients, the number of patients in both groups was 7652 patients. Finally, the recruited patients were followed-up to mortality, the new creation of AVF or AVG, or the end of the study, which was 31 December 2018.

### 2.3. Statistical Analyses

The differences in the AVF between the patients with and without a bridge were based on the demographic characteristics and the history of the disease. The χ^2^ test was used for the categorical variables (the variables were expressed as N%), and a *t*-test was used for the continuous variables (the variables were expressed as means ± standard deviation). To reduce the confounding factors, we used a 1:1 propensity score, which was estimated using a logistic regression model. Furthermore, Kaplan–Meier and Cox regression analyses were performed to analyze the new AVF or AVG creation. The results were determined as hazard ratios (HRs) with 95% confidence intervals (CIs). Adjusted HRs (aHRs) were also expressed after adjusting for CCI scores confounders. Statistical significance was considered at 2-sided *p* < 0.05. All analyses were performed using SAS, version 9.4 (SAS Institute, Cary, NC, USA).

## 3. Results

Among the patients with HD via AVF, before propensity matching, the numbers of the patients included in the groups with and without a TCC bridge differed significantly. After matching the age, sex, and comorbidities of the AVF patients with 1:1 propensity score matching, a total of 7652 patients were included in both groups. A total of 4472 (58.44%) males and 3180 (41.56%) females, with a mean age of 62.81 ± 13.42 years were included in the AVF patients with a bridge group. Meanwhile, 4492 (58.70%) males and 3160 (41.30%) females, with a mean age of 62.89 ± 12.97 years were included in the AVF patients without a bridge group. The prevalence of comorbidities, including hypertension, diabetes, hyperlipidemia, CAD, stroke, peripheral vascular disease, COPD, and cancer were also matched with a 1:1 propensity score. The patients were then stratified into three groups, according to the Charlson comorbidity index score (CCIS). The AVF patients with a bridge had a higher proportion of CCIS 3–5 (31.63% with a bridge and 28.32% without a bridge), whereas the AVF patients without a bridge had a higher proportion of CCIS 0–2 (3.41% with a bridge and 4.41% without a bridge) and CCIS ≥ 6 (64.95% with a bridge and 67.26% without a bridge). Table 1 summarizes the patient characteristics according to the presence or absence of a TCC in AVF patients before and after matching.

### Comparisons of Vascular Outcomes

The comparisons of the vascular outcomes between the AVF patients with and without a bridge during the follow-up period are presented in Table 2. Here, the vascular outcomes were regarded as the abandonment of the initial AVF that needed new AVF or new AVG creation. In patients with chronic HD, HD was maintained through the initial AVF regardless of the history of a TCC as a bridge. However, when permanent failure of the initial AVF occurred, a new AVF or a new AVG was created. Before matching the age, sex, and comorbidities, the incidence of new AVF creation was higher among the patients with a TCC bridge (0.338 event/patient-year in patients with a bridge and 0.138 event/patient-year in patients without a bridge; *p* < 0.001) and the incidence of new AVG creation was also higher among the patients with a bridge (0.146 event/patient-year in patients with a bridge and 0.041 event/patient-year in patients without a bridge; *p* < 0.001). The incidence of vascular outcomes was evaluated again after the matching of age, sex, and comorbidities. The results were consistent with those before the matching. Higher proportions of new AVF creation (0.334 event/patient-year in patients with a bridge and 0.119 event/patient-year in patients without a bridge; *p* < 0.001) and new AVG creation (0.147 event/patient-year in patients with a bridge and 0.036 event/patient-year in patients without a bridge; *p* < 0.001) were found among the patients with a bridge during the follow-up period.

The risk of new VA creation between patients on chronic HD via AVF with or without a TCC for a bridge was evaluated using Cox regression analysis, and the results are also demonstrated in Table 2. The risk of new AVF creation was higher among the dialysis patients with TCC placement (crude HR: 2.17, 95% CI: 1.77–2.66; *p* < 0.001) when compared with patients without a bridge. Similarly, the risk of AVG creation was higher among the AVF patients with a bridge than patients without a bridge (crude HR: 1.51, 95% CI: 1.15–1.98; *p* = 0.003). Therefore, the placement of a TCC as bridge therapy is a significant risk factor for AVF failure. 

**Figure 2:**
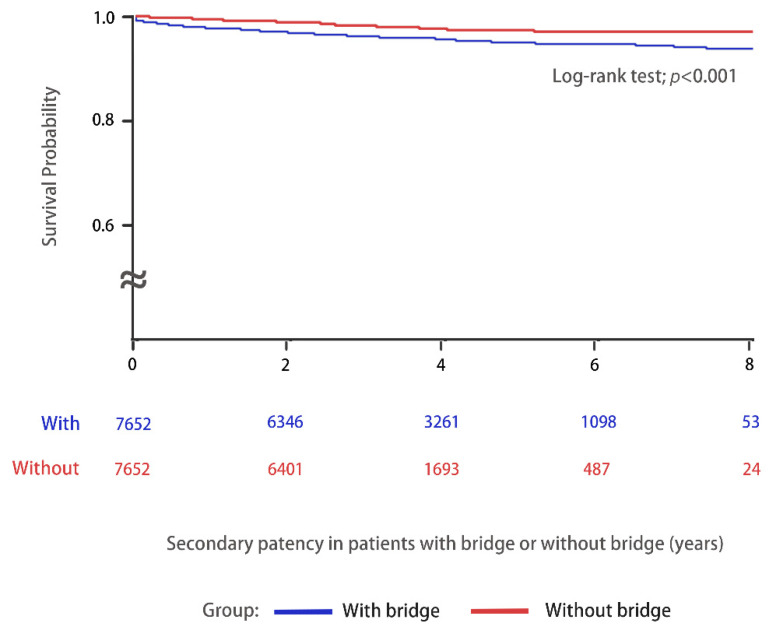
Kaplan–Meier curves were used to evaluate the secondary patency of AVF (AVF failure) with (*n* = 7652, blue line) or without (*n* = 7652, red line) a TCC as a bridge in patients on chronic HD between 1 January 2010 and 31 December 2015 and were followed-up until 31 December 2018.

Survival probability and secondary patency of the AVF during the follow-up period was compared in Figure 2. Patients on chronic HD via AVF without a TCC bridge had better survival probability and secondary patency of VA compared with those with a bridge (log-rank test; *p* < 0.001). The patients on chronic HD via AVF with and without a TCC for a bridge were subdivided into groups according to the various follow-up periods in Table 3. The incidence of new VA creation among the various groups was recorded and the results are listed. The results were analyzed by Cox regression analysis. The incidence of new AVF creation was significantly higher among the patients with a bridge than those without a bridge during the first year of follow-up. Therefore, special attention is needed to identify the signs of AVF failure during the first year of dialysis, especially in patients with a TCC as a bridge.

## 4. Discussion

The results demonstrate that the placement of a TCC as a bridge for HD was associated with a higher risk of future AVF failure and an increased risk of further AVA creation. The incidence of AVF failure and new AVA creation was highest during the first year of follow-up. Patients on HD via AVF without a history of a TCC as a bridge had better secondary patency of VA and better survival probability.

TCC for HD are widely applied in older patients because of high comorbidities, shortened life expectancy, and poor vasculature for the creation and maturation of AVF [9,10]. Increased age is a nonmodifiable factor for AVF patency [11]. In our study, the age distributions between the patients on HD with a pre-established AVF, and patients on HD with a history of a TCC as a bridge to AVF, were similar. Therefore, this factor did not interfere with the study. Patients with diabetes were more likely to experience AVF failure [12,13], but in our study, after matching the ratio of patients with diabetes between the two groups, the influence was similar, lessening this effect. Other comorbidities, such as PAD [14], that are possibly associated with AVF failure, were also adjusted for by matching.

The right internal jugular vein is the preferred VA site for TCC insertion [1,15], and more than 90% of patients on HD with a TCC were inserted on this site. According to a Canadian observational cohort study, risks for CVC-related bacteremia, malfunction, and central stenosis were 9%, 15%, and 2% at 1 year, respectively [16]. The possible mechanisms of central vein stenosis by catheters are CVC-induced trauma to the venous endothelium, and the resultant inflammatory response within the vessel wall. Other factors included the presence of a foreign body in the vein, sliding movement of the catheter with respiration, postural and head movements, increased flow, and turbulence from AVF creation, which stimulated various processes within the vessel wall and led to intimal hyperplasia of the central vein [17]. The central vein serves as an outflow tract of the AVF, and TCC-induced stenosis leads to venous stasis due to the high venous pressure. This effect persists even after the removal of the catheter [17,18,19]. Therefore, the high failure rate of AVF in patients with a history of TCC could be understood because of the hemodynamic change related to thrombosis [20]. Based on the aforementioned explanations, high AVF failure for patients on HD with a history of TCC insertion is reasonable. 

In addition to high AVF failure among patients on HD with a TCC as a bridge to AVF, our study also demonstrated the occurrence of AVF failure for patients with a history of TCC, especially during the year after the removal of the catheter. First, patients with initial HD therapy via a catheter would like to have HD via a created AVF as soon as possible because of the higher risk of catheter infection, the lower patency of the catheter, the uncomfortable feelings related to not taking a bath, and the unpleasant appearance of the catheter. Therefore, early cannulation for AVF for those patients with a TCC can be performed. The early cannulation of AVF, especially in less than 14 days, reduces AVF survival, with a 2.1-fold increased risk of subsequent fistula failure compared with fistulae cannulated after 14 days [21]. Second, the central venous catheter could result in central vein stenosis, which will reduce the chances of AVF maturation, especially if placed on the same site as a previous catheter. Pisoni et al. demonstrated that AVAs displayed better access survival if used in HD, compared with those used in HD for patients with a catheter as a bridge [22]. Third, relative to the good long-term patency of AVFs, the problem of the high prevalence of AVF maturation failure is difficult to solve. 

By contrast, advanced CKD patients, with planned AVF creation 3 to 6 months before the expected initiation of HD treatment, have enough time for the AVF to mature. For patients on HD without the planned creation of AVFs, sequential balloon-assisted maturation (BAM) could also be applied to accelerate the maturation times of the AVFs [23,24]. However, some studies have reported the negative consequences of BAM, such as fibrosis and restenosis of the venous outflow, leading to malfunctioning AVFs [25,26]. Therefore, patients on HD with planned AVF creation and without a history of TCC could have better characteristics of AVF for needling to perform HD therapy. 

The study had several limitations that may affect the interpretation of the results. First, the NHIRD does not include detailed information on smoking habits [27], the arterial or venous diameter before the creation of AVAs [28], the experiences of surgeons, the anastomosis type of the AVFs [29], the sites of AVAs, the preoperative radial artery volume flow [30], early referral for the creation of AVAs [31], and the use of far-infrared therapy [32]. These limitations may have affected the patency of the AVAs. Second, no data regarding the etiologies of the failure of AVAs are available, and we cannot know from the database if the newly created AVAs involved ipsilateral or contralateral insertion of the CVC. Third, the results from a retrospective cohort study are of a lower statistical quality compared with prospective studies. Fourth, the results might not be applicable to the populations of other ethnic backgrounds, because most of Taiwan’s population is of Chinese ethnicity. Fifth, an AVF might not fit all patients, and the ideal vascular access is the one that best suits a patient’s needs, according to the 2019 KDOQI guidelines [1]. Sixth, data on the cause of the permanent failure of AVF cannot be obtained from the NHIRD.

In conclusion, this population-based retrospective cohort study revealed that a history of TCC insertion is a risk for future AVF failure among patients on HD. Planned AVF creation and mature AVF prior to the initiation of HD is the best strategy for long-term HD. For those patients on chronic HD, with a history of TCC insertion, careful monitoring, and surveillance of AVFs are required. Further well-designed prospective clinical study will be warranted. 

## 5. Conclusions

A history of TCC was an independent risk factor for AVF failure and the time of AVF failure was significantly higher during the first year follow-up after the fistula cannulation in the TCC bridge group.

## Figures and Tables

**Table 1:** Baseline demography of chronic hemodialysis (HD) patients via arteriovenous fistulae (AVF) to maintain HD with and without a history of a tunnel cuffed catheter (TCC) as a bridge.

AVF						
Variables	Before Matching		*p*	After Matching		*p*
	With Bridge (*N* = 7670)	Without Bridge (*N* = 16,472)		With Bridge (*N* = 7652)	Without Bridge (*N* = 7652)	
**Gender**						
Male	4486 (58.49)	9453 (57.39)	0.107	4472 (58.44)	4492 (58.70)	0.742
Female	3184 (41.51)	7019 (42.61)		3180 (41.56)	3160 (41.30)	
Age stratified, y	62.79 ± 13.42	64.45 ± 12.58	<0.001	62.81 ± 13.42	62.89 ± 12.97	0.702
**Age group**						
<50	1207 (15.74)	1972 (11.97)	<0.001	1203 (15.72)	1147 (14.99)	0.390
50–64	2865 (37.35)	5982 (36.32)		2851 (37.26)	2907 (37.99)	
≥65	3598 (46.91)	8518 (51.71)		3598 (47.02)	3598 (47.02)	
**Comorbidities**						
Hypertension	4270 (55.67)	8884 (53.93)	0.011	4259 (55.66)	4320 (56.46)	0.320
Diabetes mellitus	4807 (62.67)	8687 (52.74)	<0.001	4789 (62.58)	4819 (62.98)	0.615
Hyperlipidemia	2469 (32.19)	4547 (27.60)	<0.001	2454 (32.07)	2404 (31.42)	0.385
CAD	3611 (47.08)	6403 (38.87)	<0.001	3593 (46.96)	3644 (47.62)	0.409
Stroke	2224 (29.00)	3793 (23.03)	<0.001	2208 (28.86)	2231 (29.16)	0.682
PVD	1630 (21.25)	2549 (15.47)	<0.001	1612 (21.07)	1656 (21.64)	0.385
COPD	2057 (26.82)	3319 (20.15)	<0.001	2041 (26.67)	1972 (25.77)	0.204
Cancer	49 (0.64)	130 (0.79)	0.204	49 (0.64)	51 (0.67)	0.841
**CCIS stratified**						
0–2	261 (3.40)	952 (5.78)	<0.001	261 (3.41)	338 (4.41)	<0.001
3–5	2421 (31.56)	5541 (33.64)		2421 (31.63)	2167 (28.32)	
≥6	4988 (65.03)	9979 (60.58)		4970 (64.95)	5147 (67.26)	

Data are expressed as *N* (%) for categorical variables; mean ± standard deviation for continues variables.

**Table 2:** Vascular access (VA) outcomes and Cox proportional hazards analysis for the relative risk of new AVF and new AVG creation for chronic HD patients via AVF to maintain HD with and without a history of a TCC as a bridge during the follow-up period.

**Before Matching**				
**Variables**	**With Bridge** **(*N* = 7670)**	**Without Bridge** **(*N* = 16,472)**	**Crude HR** **(95% CI)**	**Adjusted HR** **(95% CI)**
	**IR**			
New AVF creation	0.338	0.138	2.54 (2.15–3.00) ***	2.50 (2.12–2.96) ***
New AVG creation	0.146	0.041	1.69 (1.35–2.12) ***	1.65 (1.32–2.07) ***
**After Matching**				
**Variables**	**With Bridge** **(*N* = 7652)**	**Without Bridge** **(*N* = 7652)**	**Crude HR** **(95% CI)**	**Adjusted HR** **(95% CI)**
	**IR**			
New AVF creation	0.334	0.119	2.17 (1.77–2.66) ***	2.17 (1.77–2.67) ***
New AVG creation	0.147	0.036	1.51 (1.15–1.98) **	1.52 (1.15–1.99) **

IR—Incidence rate; HR—Hazard ratio; **: *p* < 0.01; ***: *p* < 0.001.

**Table 3:** Comparison of incident times of new AVF and new AVG creation between patients on chronic HD via AVF to maintain HD with and without TCC for bridges.

New AVF Creation						New AVG Creation				
Variables (Years)	With Bridge (*N* = 7661)		Without Bridge (*N* = 7661)		*p*-Value	With Bridge (*N* = 7661)		Without Bridge (*N* = 7661)		*p*-Value
	*n*	PY	*n*	PY		*n*	PY	*n*	PY	
0 ≤ Follow up time < 1	72	7.849	14	8.492	<0.001	44	14.967	18	9.218	0.143
1 ≤ Follow up time < 2	20	31.663	12	17.215	0.787	26	39.961	13	18.436	0.812
2 ≤ Follow up time < 3	17	41.590	12	28.807	0.959	16	39.148	13	31.181	0.957
Follow up time ≥ 3	19	79.808	6	25.032	0.988	25	107.356	10	40.301	0.865

*n* = event number; PY = patient-years.

## Data Availability

According to the General Data Protection Regulation, the data presented in this research are not publicly available.

## References

[1] Lok C.E., Huber T.S., Lee T., Shenoy S., Yevzlin A.S., Abreo K., Allon M., Asif A., Astor B.C., Glickman M.H. (2020). KDOQI Clinical Practice Guideline for Vascular Access: 2019 Update. Am. J. Kidney Dis..

[2] Almasri J., Alsawas M., Mainou M., Mustafa R.A., Wang Z., Woo K., Cull D.L., Murad M.H. (2016). Outcomes of vascular access for hemodialysis: A systematic review and meta-analysis. J. Vasc. Surg..

[3] Celik S., Gok O.E., Ulusal O.G., Selen T., Ayli M.D. (2021). The impact of arteriovenous fistulas and tunneled cuffed venous catheters on morbidity and mortality in hemodialysis patients: A single center experience. Int. J. Artif. Organs..

[4] Shechter S.M., Skandari M.R., Zalunardo N. (2014). Timing of arteriovenous fistula creation in patients With CKD: A decision analysis. Am. J. Kidney Dis..

[5] Schmidli J., Widmer M.K., Basile C., de Donato G., Gallieni M., Gibbons C.P., Haage P., Hamilton G., Hedin U., Kamper L. (2018). Editor’s Choice—Vascular Access: 2018 Clinical Practice Guidelines of the European Society for Vascular Surgery (ESVS). Eur. J. Vasc. Endovasc. Surg..

[6] Vassalotti J.A., Jennings W.C., Beathard G.A., Neumann M., Caponi S., Fox C.H., Spergel L.M. (2012). Fistula First Breakthrough Initiative Community Education Committee. Fistula first breakthrough initiative: Targeting catheter last in fistula first. Semin. Dial..

[7] Lee T. (2017). Fistula First Initiative: Historical Impact on Vascular Access Practice Patterns and Influence on Future Vascular Access Care. Cardiovasc. Eng. Technol..

[8] Taiwan Society of Nephrology (2019). Annual Report on Kidney Disease in Taiwan.

[9] Canaud B., Tong L., Tentori F., Akiba T., Karaboyas A., Gillespie B., Akizawa T., Pisoni R.L., Bommer J., Port F.K. (2011). Clinical practices and outcomes in elderly hemodialysis patients: Results from the Dialysis Outcomes and Practice Patterns Study (DOPPS). Clin. J. Am. Soc. Nephrol..

[10] Al-Jaishi A.A., Oliver M.J., Thomas S.M., Lok C.E., Zhang J.C., Garg A.X., Kosa S.D., Quinn R.R., Moist L.M. (2014). Patency rates of the arteriovenous fistula for hemodialysis: A systematic review and meta-analysis. Am. J. Kidney Dis..

[11] Lazarides M.K., Georgiadis G.S., Antoniou G.A., Staramos D.N. (2007). A meta-analysis of dialysis access outcome in elderly patients. J. Vasc. Surg..

[12] Yan Y., Ye D., Yang L., Ye W., Zhan D., Zhang L., Xiao J., Zeng Y., Chen Q. (2018). A meta-analysis of the association between diabetic patients and AVF failure in dialysis. Ren. Fail..

[13] Sedlacek M., Teodorescu V., Falk A., Vassalotti J.A., Uribarri J. (2001). Hemodialysis access placement with preoperative noninvasive vascular mapping: Comparison between patients with and without diabetes. Am. J. Kidney Dis..

[14] Chen S.C., Chang J.M., Hwang S.J., Tsai J.C., Wang C.S., Mai H.C., Lin F.H., Su H.M., Chen H.C. (2009). Significant correlation between ankle-brachial index and vascular access failure in hemodialysis patients. Clin. J. Am. Soc. Nephrol..

[15] Work J. (2002). Hemodialysis catheters and ports. Semin. Nephrol..

[16] Poinen K., Quinn R.R., Clarke A., Ravani P., Hiremath S., Miller L.M., Blake P.G., Oliver M.J. (2019). Complications from tunneled hemodialysis catheters: A Canadian observational cohort study. Am. J. Kidney Dis..

[17] Agarwal A.K., Patel B.M., Haddad N.J. (2007). Central vein stenosis: A nephrologist’s perspective. Semin. Dial..

[18] MacRae J.M., Ahmed A., Johnson N., Levin A., Kiaii M. (2005). Central vein stenosis: A common problem in patients on hemodialysis. ASAIO J..

[19] Forauer A.R., Theoharis C. (2003). Histologic changes in the human vein wall adjacent to indwelling central venous catheters. J. Vasc. Interv. Radiol..

[20] Shingarev R., Barker-Finkel J., Allon M. (2012). Association of hemodialysis central venous catheter use with ipsilateral arteriovenous vascular access survival. Am. J. Kidney Dis..

[21] Rayner H.C.R., Pisoni R.L., Gillespie B.W., Goodkin D.A., Akiba T., Akizawa T., Saito A., Young E.W., Port F.K. (2003). Creation, cannulation and survival of arteriovenous fistulae: Data from the Dialysis Outcomes and Practice Patterns Study. Kidney Int..

[22] Pisoni R.L., Young E.W., Dykstra D.M., Greenwood R.N., Hecking E., Gillespie B., Wolfe R.A., Goodkin D.A., Held P.J. (2002). Vascular access use in Europe and the United States: Results from the DOPPS. Kidney Int..

[23] Lee T., Ullah A., Allon M., Succop P., El-Khatib M., Munda R., Roy-Chaudhury P. (2011). Decreased cumulative access survival in arteriovenous fistulas requiring interventions to promote maturation. Clin. J. Am. Soc. Nephrol..

[24] Elkassaby M., Elsaadany N., Mowaphy K., Soliman M. (2021). Balloon-assisted maturation of autogenous arteriovenous fistulae: A randomized controlled prospective study. Vascular.

[25] Rizvi S.A., Usoh F., Hingorani A., Iadgarova E., Boniscavage P., Eisenberg J., Ascher E., Marks N. (2017). The clinical efficacy of balloon-assisted maturation of autogenous arteriovenous fistulae. Ann. Vasc. Surg..

[26] Peterson W.J., Barker J., Allon M. (2008). Disparities in fistula maturation persist despite preoperative vascular mapping. Clin. J. Am. Soc. Nephrol..

[27] Monroy-Cuadros M., Yilmaz S., Salazar-Bañuelos A., Doig C. (2010). Risk factors associated with patency loss of hemodialysis vascular access within 6 months. Clin. J. Am. Soc. Nephrol..

[28] Li H.L., Chan Y.C., Cui D., Liu J., Wang M., Li N., Pai P., Cheng S.W. (2020). Predictors of primary functional maturation of autogenous radiocephalic arteriovenous fistula in a cohort of Asian patients. Ann. Vasc. Surg..

[29] Bashar K., Conlon P.J., Kheirelseid E.A., Aherne T., Walsh S.R., Leahy A. (2016). Arteriovenous fistula in dialysis patients: Factors implicated in early and late AVF maturation failure. Surgeon.

[30] Masengu A., McDaid J., Maxwell A.P., Hanko J.B. (2016). Preoperative radial artery volume flow is predictive of arteriovenous fistula outcomes. J. Vasc. Surg..

[31] Weber C.L., Djurdjev O., Levin A., Kiaii M. (2009). Outcomes of vascular access creation prior to dialysis: Building the case for early referral. ASAIO J..

[32] Lindhard K., Rix M., Heaf J.G., Hansen H.P., Pedersen B.L., Jensen B.L., Hansen D. (2021). Effect of far infrared therapy on arteriovenous fistula maturation, survival and stenosis in hemodialysis patients, a randomized, controlled clinical trial: The FAITH on fistula trial. BMC Nephrol..

